# Potassium Radioisotope 40 as Component of Mitochondria Physiology: Therapy Proposal for Mitochondrial Disfunction Diseases

**DOI:** 10.3389/fpubh.2020.578392

**Published:** 2020-10-09

**Authors:** Maurizio Tomasi

**Affiliations:** Department of Biology, New Mexico State University, Las Cruces, NM, United States

**Keywords:** background ionizing radiations, Auger electrons, electron transport chain, mitochondria, hydrogen peroxide signaling, Coenzyme Q_10_, mitochondrial dysfunction

## Introduction

Huge quantities of energy poured onto the still-crusting earth. Solar storms, supernova explosions, volcanic eruptions, geysers were very frequent events in the Archaeozoic period, 3.5·10^9^ years ago. A melting pot of electrons, protons, free radicals, methane, ammonia, hydrogen, water, and carbon dioxide whose meeting and collision has produced billions of compounds. And together they made up the primordial soup. A broth where matter aggregated into orderly structures that allowed life and its expansion. Alternative to the primordial soup, the iron-sulfur world hypothesis has been advanced: the mineral surfaces of iron sulphides provided the catalytic conditions for giving rise to life (1). However both hypothesis can be complementary if the ocean had been a relatively small sea portion. The slow depletion of the nutrients selected the cyanobacteria, which thanks to the chlorophyll synthesis required only a few very common elements to reproduce: sun, water, and minerals. The expanding plant world has provided the nutrients which allowed the birth of the animal world. The environment and the living organisms changed each other. The atmosphere turned into oxidative from a reducing agent. Through the chlorophyll synthesis solar energy has been transformed in chemical bonds so that all the anabolism and catabolism were determined by energy exchanges provided by electron and proton movements. Among all types of natural ionizing radiation (NIR), which scattered the energy of electrons and protons in all directions, light radiations have become the energy unique source by channeling electrons and protons on strictly defined paths. Life has grown in complexity and refinement, and this has kept the memory of the previous state of complexity (2). For example, the common characteristics of the electron transport chains of mitochondria and chloroplasts represent the inheritance received from the same ancestor.

Currently, NIR refers only to ionizing radiation from the cosmos and radioactive minerals, which are toxic in high concentrations causing irreversible genetic and metabolic damage. Toxicity is related to the average of the energy deposited in the recipient organism (energy defined as LET, Linear Energy Transfer). Initially the toxicity of the NIR on the earth's surface was predicted by extrapolating LET. Therefore, even small doses of NIR should have been harmful, and if an organism had managed to grow in an environment with reduced quantities of NIR, the organism would have had to benefit from it. The hypothesis was first tested by shielding Paramecium Tetraurelia cultures with lead plates (3). Subsequently prof. Luigi Satta, with a brilliant intuition, promoted the construction of a biology section in the National Nuclear Physics laboratories inside the Gran Sasso tunnel (LNGS). The results showed that the NIR at surface level are stimulating rather than inhibiting, showing a two-phase trend of hormesis, i.e., linear only in high energy (4, 5). The effect does not seem due to the involvement of genetic mutation, since an evolutionary experiment on *E. coli* cultures followed for 500 generations both in surface (SL) and underground (UL) laboratories, excludes it (6). Other research has indicated that NIR regulates metabolic homeostasis. A study on *Drosophila melanogaster* showed less fertility and greater longevity when midges grow in an underground laboratory (UL) than those grown on the surface (SL) (7). Data from *Shewanella oneidensis* transcriptome analysis also suggest that NIR are involved in metabolic homeostasis. In fact, UL cultures show a marked decrease in ribosomal protein synthesis, and in respiratory chain proteins as well (8). However, in energy terms, not all radiation has the same toxicity. Photons at 20 KeV are not toxic as a dose of ray x at 20 KeV. And ray x at 20 KeV affect quite differently a *Deinococcus radiodurans* or one belonging to the Flavobacterium species. The former is an extremophilic bacterium that resists cold, dehydration, vacuum, and even an acidic environment, on the contrary the latter is sensitive to any environmental variations.

At odd there are bacteria as *Candidatus Desulforudis audaxviator*, which lives in areas without light and obtain energy from the radioactivity of Uranium, Thorium and Potassium (9).

## Discussion

The use of toxic radiations as a source of energy, suggested to review the data analysis on the radioisotope 40 of Potassium (^40^K) which prompted Moore and Sastry (10) to speculate that ^40^K was the “primordial gene irradiator.” Hypothesis which was later discarded as reported in (11). However, the analysis on the possible biological role of ^40^K, performed by Moore and Sastry, has such consistency as to suggest that it is worth considering an alternative biological role with respect to the mutagenetic one. The hypothesis, presented here, considers that the beta decays of ^40^K could rather play an energetic role, which, although tiny, could intervene in very special situations. It is important to stress that ^40^K beta decay includes the Auger and Coster-Kronig electrons (ACKE), which travel few nanometers distances and can be deposited on molecules very close to K. The ACKE cannot be measured as ambient radioactivity because of their very low energy content, still these beta decays are the most effective, thanks to the drastic changes induced on molecules acquiring an electron outside their metabolic context.

Actually the ^40^K is the main source of radioactivity within living organisms. In the early stages of evolution, about 3.5·10^9^ years ago, it was seven times higher than now, but likely has been well-tolerated or even integrated into living organisms. The maintenance of unbalanced concentrations is guaranteed by specific channels for K (Kcls). Inside the mitochondria Kcls finely modulate apoptosis, respiratory activity, volume, signal transmission and anti-stress action.

To date there is no data on ACKE target molecules, therefore on the matter there is wide space for speculations. If oxygen O_2_ were a target molecule, superoxide is formed, which before and immediately afterwards, would spawn hydrogen peroxide (H_2_O_2_). A chemical compound that in small concentration, acts as signaling molecule in a wide variety of processes, spanning from embryonic development to cell death, and in the nervous system can modulate both neuronal excitability and dopamine neuronal firing (12). Since H_2_O_2_ rapidly diffuses because of its membrane permeability, its signals must act in fractions of a second (13). Therefore, opening and closing of the Kcls could modulate both timing and sites of the H_2_O_2_ production through dynamic gradient of ^40^K.

Another hypothesis predicts that ^40^K is somehow involved in the mitochondria respiratory chain. The rationale is as follows: K with its isotope ^40^K, 3.5 billion years ago, had a radioactivity seven times greater than the present time, the continuous and conspicuous emission of ACKE could therefore have contributed to the construction of a primitive transport chain. Over the course of time ^40^K has gradually lost the power of radioactivity and its role has become residual, but always able to contribute to the functioning of the mitochondrial respiratory chain in restricted and determined circumstances. In addition there is another molecule of the mitochondria, which like K is practically ubiquitous in all living organisms: the ubiquinone, also known as Coenzyme Q_10_ (CoQ_10_), which play a crucial role in electronic transport. In fact, CoQ_10_ is an amphoteric lipid molecule that with its great mobility transfers electrons between complexes I, II and III. Complexes which are proteins with the tendency to form rather rigid structures unable to get electrons without a proper provider. In mitochondria, CoQ_10_ also acts as an antioxidant and as free radical scavenger. Thus CoQ_10_ protects lipid integrity, in particular that of cardiolipin, from attack by free radicals (14). A role that other antioxidants, such as glutathione or manganese superoxide dismutase, would not be able to play being in mitochondrial matrix (15). In addition CoQ_10_ membrane localization makes it close to K, precisely where its concentration is higher. It is therefore probable that part of ACKE are captured by CoQ_10_ and recycled in the respiratory chain. Under this visual angle the ACKE would supply a constant, albeit minuscule, amount of energy to the electronic transport system. However, this energy, more similar to a spark than a flame, could be useful when the respiratory chain slows down drastically, or even stops for physiological or pathological reasons, such as in the transient cases of hypoxia and lack of nutrients. In these situations the ACKE would autonomously provide those electrons which could avoid the consequences of a complete stop of mitochondrial activity. Something that should behave like the very tiny flame, constantly lit, of a switched off boiler, which, on command, allows it to be ignited.

Even if ^40^K will prove irrelevant to biological systems, the idea that ^40^K can supply electrons to CoQ_10_ could have important application in medicine, for the following reasons:

If CoQ_10_ were given in combination with ^40^K enriched K, the increase in available ACKE, once captured and transported by CoQ_10_ into the respiratory chain, should have to improve the energy efficiency of the mitochondrial system. And significant, if not synergistic, therapeutic effects should be observed. The list of pathologies caused by mitochondrial dysfunctions is quite long, only a few are mentioned here: epilepsy, aging and degenerative diseases of the nervous system. It is important to stress that CoQ_10_ is already on the market as widely established dietary supplement, which has been shown to be beneficial in a variety of clinical cases: recovery after heart failure, in treating statin myopathy, in neurological disorders, aging (16). To give an extra spurt to mitochondria should be the very reason, worthy to try, for using the CoQ_10_—K enriched ^40^K. Finally it should be emphasized that to understand the importance of ^40^K in biology there is no other way than to develop research lines that foresee the use in the culture media of K without ^40^K, but also in parallel with K enriched with ^40^K. If the experiments were carried out in underground biological laboratories, such as the WIPP in Carlsbad, New Mexico, USA, which are shielded from environmental NIR, an environment almost completely free of radioactivity would be obtained. The experimental conditions would be suitable for evaluating not only the contribution of the ^40^K but also that of the NIR on living organisms.

## Conclusions

In this work, the hypothesis is advanced that the K isotope 40 plays a role in cellular homeostasis. In particular, its beta radiations, which includes low energy electrons with high ionization potential, ACKE, could produce micro quantities of H_2_O_2_ and directly and indirectly supply electrons to the mitochondrial respiratory chain. CoQ_10_ would be responsible for the electronic transfer, which in addition to having the role of transfering electrons in the respiratory chain also has the role of free radical scavenger. Although the amounts of energy produced through ACKE appear insignificant in situations of normal metabolism, their production in the event of temporary blockage of mitochondrial activity could avoid irreversible damage and therefore help recovery.

The hypothesis has the non-secondary purpose of stimulating ^40^K research and its effects in biology and medicine.

It is important to underline that the rationale behind a scientific research proposal has been presented here: the purpose of the research is to determine whether the combined administration of CoQ_10_ and potassium enriched with radioisotope 40 can be used in the therapy of patients suffering from diseases caused by mitochondria dysfunctions.

## Author Contributions

The author confirms being the sole contributor of this work and has approved it for publication.

## Conflict of Interest

The author declares that the research was conducted in the absence of any commercial or financial relationships that could be construed as a potential conflict of interest.
